# Impact of Preservative-Free Travoprost on Intraocular Pressure and Ocular Surface in Primary Open-Angle Glaucoma Patients

**DOI:** 10.3390/life15121870

**Published:** 2025-12-06

**Authors:** Minas Bakirtzis, Panagiota Ntonti, Eirini-Kanella Panagiotopoulou, Aristeidis Konstantinidis, Vangelis G. Manolopoulos, George Kolios, Georgios Labiris

**Affiliations:** 1 Department of Ophthalmology, University Hospital of Alexandroupolis, Democritus University of Thrace, Dragana Campus, 68100 Alexandroupolis, Greece; giotado@hotmail.com (P.N.); panagiotopoulou.ek@gmail.com (E.-K.P.); aristeidiskon@hotmail.com (A.K.); 2 Laboratory of Pharmacology, Faculty of Medicine, Democritus University of Thrace, 68100 Alexandroupolis, Greece; emanolop@med.duth.gr (V.G.M.); gkolios@med.duth.gr (G.K.); 3 Individualised Medicine & Pharmacological Research Solutions Center (IMPReS), Democritus University of Thrace, 68100 Alexandroupolis, Greece

**Keywords:** travoprost, primary open-angle glaucoma, ocular surface, preservatives

## Abstract

Background: The present study assesses the effectiveness and tolerability of preservative-free travoprost in patients with primary open-angle glaucoma (POAG), focusing on intraocular pressure (IOP) reduction and ocular surface health over a six-month period. Methods: The study was prospectively designed and conducted at the University General Hospital of Alexandroupolis, Greece. A total of 45 patients diagnosed with either POAG or ocular hypertension (OHT) were included in the study; all were either newly diagnosed or had previously discontinued prostaglandin therapy due to intolerance. Of these, 39 participants completed the study. All subjects were administered preservative-free travoprost once daily. Clinical evaluations were conducted at baseline (T0), and at 1 month (T1), 3 months (T3), 6 months (T6) and 12 months (T12) following treatment initiation. The primary outcomes encompassed reductions in intraocular pressure (IOP), Schirmer test values, tear break-up time (TBUT), conjunctival hyperemia, as well as measurements of retinal nerve fiber layer (RNFL) and ganglion cell complex (GCC) thickness. Visual field parameters were also assessed. Results: A significant reduction in IOP was observed at T1, T3, T6 and T12 compared to baseline (*p* < 0.01). Schirmer test scores improved significantly from T0 to all subsequent time points (*p* < 0.01). Conjunctival hyperemia decreased significantly across follow-ups, while TBUT showed no significant change (*p* > 0.05). No significant changes were noted in mean deviation (MD), pattern standard deviation (PSD), RNFL, or GCC thickness over six months. Conclusion: Preservative-free travoprost effectively reduces IOP while improving ocular surface health, particularly in tear production and conjunctival hyperemia.

## 1. Introduction

Glaucoma, a progressive optic nerve disorder, is among the top causes of irreversible blindness, affecting over 76 million people globally—a figure expected to rise to 111.8 million by 2040 [1,2]. Glaucoma can be classified into several types, with primary open-angle glaucoma (POAG) being the most prevalent form. POAG is characterized by an increase in intraocular pressure (IOP), resulting in optic nerve degeneration and subsequent visual impairment [3]. Among the risk factors for POAG, elevated IOP is the most significant and the only one that can currently be modified through treatment [3].

Recent epidemiological analyses indicate that the global prevalence of POAG continues to rise, with approximately 57.5 million individuals affected worldwide in 2020 and projections estimating more than 100 million cases by 2040, disproportionately affecting Asian and African populations [1,2]. This growing prevalence underscores the importance of optimizing therapeutic strategies that effectively lower intraocular pressure while minimizing treatment-related adverse effects [3].

The principal objective of glaucoma management is the reduction in IOP, thereby preventing or slowing further optic nerve damage [4,5,6]. Pharmacological therapy remains the first-line treatment for POAG, with prostaglandin analogs being the most frequently prescribed class of drugs due to their efficacy in lowering IOP [4,7,8,9]. Travoprost, a prostaglandin F2-alpha analog, enhances aqueous humor outflow via both the trabecular meshwork and the uveoscleral pathway [10,11,12,13].

Although prostaglandin analogs are highly effective in lowering intraocular pressure, many formulations include preservatives like benzalkonium chloride (BAK), which are used to prevent microbial growth and prolong shelf life [14,15,16]. However, preservatives such as benzalkonium chloride (BAK) have been linked to a range of ocular surface side effects, especially in patients receiving long-term therapy [17,18]. Long-term exposure to BAK may cause ocular surface disease (OSD), presenting symptoms such as dry eye syndrome, conjunctival hyperemia, damage to the corneal epithelium, and instability of the tear film. OSD not only reduces quality of life for patients but can also lead to poor adherence to treatment and subsequent glaucoma progression [19].

BAK is a cationic surfactant commonly used as a preservative in ophthalmic preparations. Its surface-active properties can interact with lipid components at the aqueous–lipid interface and, in vitro and in vivo, have been associated with reduced tear-film stability. However, BAK’s clinically important effects are multifactorial: direct epithelial cytotoxicity, induction of subclinical inflammation, and loss of conjunctival goblet cells with consequent reduction in mucin production—all of which impair tear-film homeostasis and promote evaporative dry eye. Clinically, these mechanisms contribute to tear film instability, conjunctival hyperemia, and corneal epitheliopathy, which not only worsen ocular surface disease but also reduce patient adherence and tolerance to therapy. The clinical relevance of minimizing or eliminating BAK exposure is therefore particularly significant in glaucoma patients requiring lifelong treatment [20,21].

In response to the growing impact of preservative-induced ocular surface toxicity, preservative-free formulations of prostaglandin analogs, including travoprost, have been developed [19,20,21,22]. These preservative-free formulations aim to reduce the risk of OSD while maintaining the IOP-lowering effects of the medication [19,22,23,24].

Travoprost was selected for this study because, among prostaglandin analogues, it has demonstrated robust IOP-lowering efficacy through dual mechanisms of action (enhancing both trabecular and uveoscleral outflow). Previous formulations of travoprost contained BAK or alternative preservatives, both of which may still exert ocular surface toxicity. Evaluating a completely preservative-free travoprost formulation therefore provides an opportunity to determine whether the proven efficacy of travoprost can be maintained while eliminating the risks associated with preservatives. This distinction sets it apart from other prostaglandins, where preservative-free options have been more extensively investigated [25,26,27].

While earlier studies have primarily investigated BAK-free travoprost formulations containing alternative preservatives such as polyquaternium-1 or SofZia^®^ (Novartis AG, Basel, Switzerland), there is limited evidence regarding formulations completely devoid of preservatives. To our knowledge, the present study is the first to specifically evaluate the dual impact of a preservative-free travoprost formulation on both intraocular pressure control and ocular surface health in patients with primary open-angle glaucoma. By focusing on these complementary outcomes, this work seeks to address an important gap in the literature and provide new evidence supporting the clinical value of preservative-free prostaglandin therapy [25,26,27].

Within this context the current study seeks to assess the efficacy and safety of preservative-free travoprost in a group of patients with POAG, focusing on both IOP reduction and improvements in ocular surface.

## 2. Materials and Methods

### 2.1. Study Design

This prospective observational study was conducted to assess the efficacy and safety of preservative-free travoprost in patients with POAG. It aimed to evaluate the IOP-lowering effect of preservative-free travoprost while monitoring its impact on ocular surface health over a twelve-month period. The study complied with the principles of the Declaration of Helsinki, and all participants provided written informed consent before enrollment. The study protocol was approved by the Institutional Review Board of the University Hospital of Alexandroupolis in Greece, where the research took place (protocol code ES3/03-02-2022 and date of approval 3 February 2022). ClinicalTrials.gov identifier: NCT05319470.

### 2.2. Study Population

Patients were recruited from the outpatient service of the Department of Ophthalmology of the University General Hospital of Alexandroupolis. Inclusion criteria consisted of (i) patients with a diagnosis of POAG or ocular hypertension (OHT) who were either treatment-naive or had discontinued previous prostaglandin medications due to intolerance for at least 2 weeks, (ii) central corneal thickness (CCT) ≥ 500 μm and CCT ≤ 600 μm and (iii) in both eyes 22 mmHg ≤ IOP < 34 mmHg. This range was chosen because values below 22 mmHg may not reliably indicate pathological elevation requiring pharmacological intervention, whereas values above 34 mmHg are generally considered advanced or at high risk of rapid progression, making such patients less suitable for short-term safety and tolerability evaluation. Central corneal thickness (CCT) between 500 μm and 600 μm was required to minimize the confounding effect of corneal thickness on Goldmann applanation tonometry readings, as thinner corneas can lead to underestimation and thicker corneas to overestimation of IOP. This restriction ensured greater accuracy and consistency of IOP measurements within the study population. Exclusion criteria included patients, (i) with advanced visual field loss (mean deviation (MD) < −18 dB), a total defect (complete loss of visual sensitivity within the tested area) within the central 10 degrees of the visual field, and a potential risk of further visual field deterioration based on the investigator’s assessment for study eligibility, (ii) with significant worsening of the 2 last visual fields (6 months between them), (iii) with psychiatric conditions, (iv) receiving additional anti-glaucoma medications, (v) with a history of allergic reactions to similar medications, and (vi) with recent ocular surgery. All patients underwent a complete slit-lamp examination to ensure corneal integrity. Subjects with active or chronic corneal disease (e.g., keratitis, keratoconjunctivitis sicca, corneal dystrophies or ectasias, scarring) or history of corneal surgery were excluded, in order to prevent confounding factors affecting ocular surface evaluation or accuracy of IOP measurements.

### 2.3. Treatment Protocol

Patients received preservative-free travoprost (Trav-IOP, Cooper S.A., Athens, Greece) as a monotherapy once daily, administered in the evening. The preservative-free travoprost used was designed to reduce the potential for ocular surface irritation and toxicity while maintaining the IOP-lowering effects of the medication.

### 2.4. Assessment and Follow-Up

All patients were examined at five key time points: baseline prior to initiating treatment (T0), and subsequently at one (T1), three (T3), six (T6) months and twelve (T12) months following the start of preservative-free travoprost therapy. At each visit, several standardized clinical parameters were assessed to monitor treatment efficacy and ocular surface status.

Intraocular pressure (IOP) was measured using Goldmann applanation tonometry, widely regarded as the gold standard in IOP assessment for accuracy and reproducibility in clinical research, thereby allowing reliable comparisons with previous glaucoma trials. For each eye, three consecutive readings were obtained, and the mean value was recorded. To ensure consistency across visits, IOP measurements were performed at the same time of day (in the morning). The primary outcome of the study was the reduction in IOP over time, with measurements documented at T0, T1, T3, T6, and T12.

Tear production was evaluated through the Schirmer test, performed using filter paper strips inserted in the lower eyelid following instillation of tetracaine 0.5%. After five minutes, the length of wetting was measured, with a value below 10 mm considered indicative of tear deficiency. Schirmer testing was performed with topical anesthesia to suppress reflex tearing, thereby providing a more accurate measure of basal tear secretion. This test was administered at each follow-up point: T0, T1, T3, T6, and T12.

Tear film stability was assessed using the Tear Break-Up Time (TBUT) test. TBUT was evaluated using fluorescein dye instilled in the inferior conjunctival sac, and the interval between a complete blink and the first appearance of a dry spot on the corneal surface was recorded under slit-lamp examination. This standardized approach was selected, as it represents the most commonly accepted method for assessing tear film stability in clinical practice. A TBUT value of less than 10 s was interpreted as indicative of tear film instability. As with other assessments, TBUT was measured at T0, T1, T3, T6, and T12.

To evaluate ocular surface inflammation, conjunctival hyperemia was graded using the validated Efron scale, ranging from 0 (normal) to 4 (severe redness). Grades were assigned as follows: 0 = normal, 1 = trace, 2 = mild, 3 = moderate, and 4 = severe. Each patient was evaluated at all four time points. This scale is publicly available for educational and clinical use. No copyright permission is required [23].

Structural integrity of the optic nerve was monitored using spectral-domain Optical Coherence Tomography (OCT) imaging with the Avanti system (Optovue Inc., Fremont, CA, USA). Retinal nerve fiber layer (RNFL) and ganglion cell complex (GCC) thickness were measured at baseline (T0), at the six-manth follow-up (T6) and at the final follow-up to detect any glaucomatous progression.

Visual field testing was performed using the Humphrey automated perimeter (Carl Zeiss Meditec, Inc., Dublin, CA, USA), employing the Swedish Interactive Threshold Algorithm (SITA) Standard with the 24-2 program. These assessments were conducted at baseline, at six months and at the final follow-up (T0, T6 and T12) to monitor functional changes related to glaucoma progression.

### 2.5. Statistical Analysis

Statistical analysis was performed using the Medcalc 20.1.4 statistical software package (MedCalc Software, Ostend, Belgium). A priori power analysis was performed to determine the necessary sample size, indicating that 28 patients were needed to complete the study in order to achieve power 80% at a 0.05 significance level with effect size at 0.23. For normally distributed repeated measures, ANOVA for repeated measures was applied, whereas the Friedman test was used for non-normally distributed repeated measures. Paired-sample t-tests were employed for comparisons between two time points. *p*-values < 0.05 were regarded as statistically significant. Missing data from patients who did not complete all follow-ups were excluded from the longitudinal analyses (complete-case analysis).

## 3. Results

### 3.1. Patient Demographics

Out of the 45 patients initially enrolled in the study, 3 patients developed an allergic reaction to the medication and withdrew from the study and 3 lost one of their follow-ups, 39 completed the full twelve-month follow-up period. A total of 78 eyes were examined over a period of 12 months following the initiation of treatment with preservative-free travoprost. Demographic data of the patients recruited in the study are presented in Table 1.

### 3.2. Intraocular Pressure (IOP)

**Figure 1:**
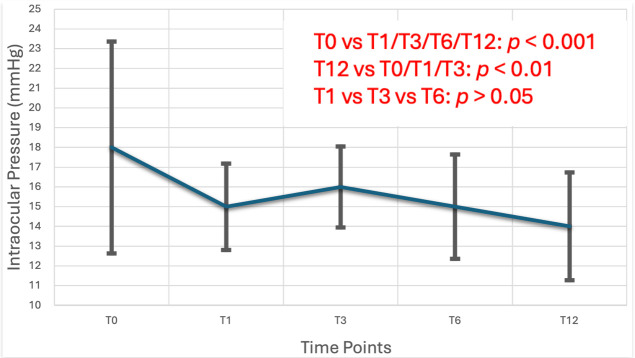
Mean intraocular pressure (IOP) reduction IOP values were recorded at baseline and at 1, 3, 6 and 12 months post-treatment initiation. A statistically significant reduction in IOP was observed over the 12-month follow-up period, indicating the efficacy of preservative-free travoprost as monotherapy.

A Friedman Test revealed a statistically significant reduction in IOP over time (x^2^(4) = 43, *p* < 0.001). Pairwise comparisons showed significant reductions in mean IOP from baseline (T0) to all subsequent visits (T1, T3, T6, and T12 *p* < 0.001), with no significant differences between T1, T3, and T6 (*p* > 0.05), indicating a sustained IOP-lowering effect. Significant differences were also detected between T12 and T0,T1 and T3 timelines (*p* < 0.01). Median baseline IOP was 18 [15, 20] mmHg, which decreased to 14 [12.8, 15] mmHg at 12 months, corresponding to a mean reduction of 4 mmHg (approximately 22.2% decrease from baseline; *p* < 0.001). These findings are illustrated in Table 2 and Figure 1.

### 3.3. Ocular Surface Parameters

**Figure 2:**
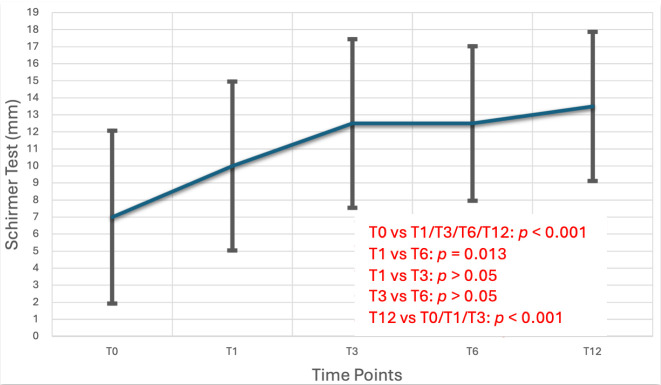
Schirmer Test scores (with anesthesia) demonstrating tear production fluctuations during the treatment period. Measurements were taken at baseline and at 1, 3, 6, and 12 months to assess the impact of the medication on aqueous tear secretion. A gradual improvement was noted, suggesting better ocular surface tolerance with preservative-free formulations.

**Figure 3:**
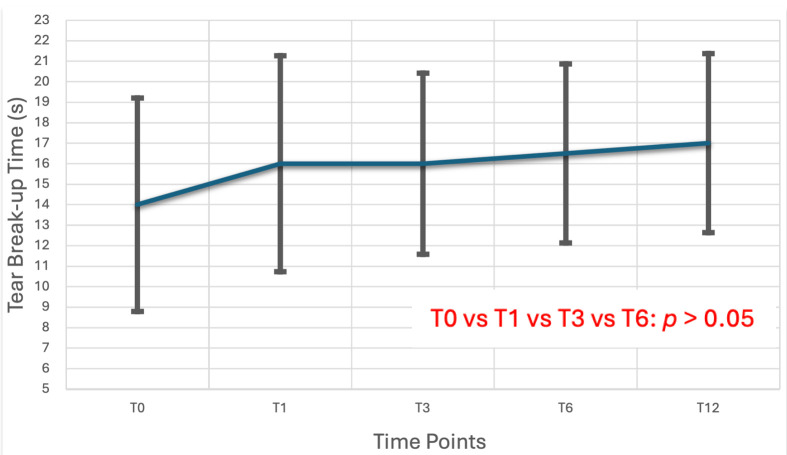
Tear Break-Up Time (TBUT) values at the same time intervals, indicating the stability of the tear film. TBUT improvement over time reflects enhanced tear film quality and reduced ocular surface irritation, supporting the safety profile of preservative-free travoprost in long-term use.

**Figure 4:**
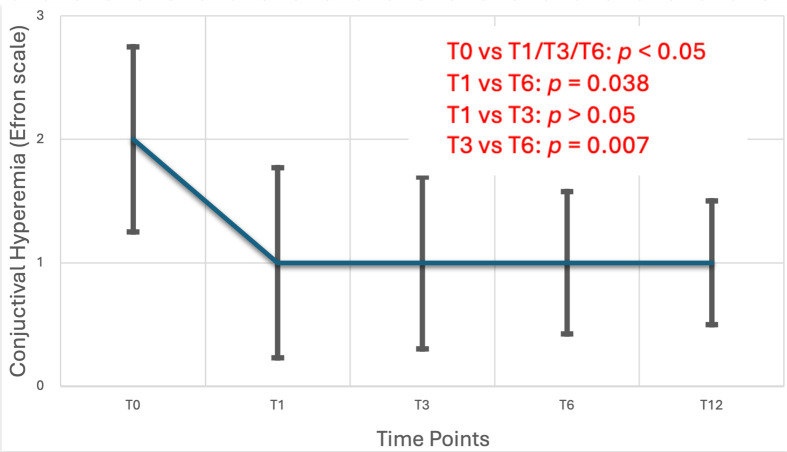
Conjunctival hyperemia grading assessed using a standardized Efron’s scale, at each follow-up point. A reduction in hyperemia scores indicates a decline in ocular surface inflammation and improved tolerability, which is clinically relevant for patients requiring chronic treatment for glaucoma. Conjunctival hyperemia was graded using the Efron Scale [28], which is publicly available for educational and clinical use. No copyright permission is required.

Statistical comparisons across the follow-up period demonstrated notable improvements in ocular surface health after the initiation of preservative-free travoprost. Tear production, as assessed by the Schirmer test, showed a statistically significant increase over time, with Friedman Test revealing a significant main effect (x^2^(3) = 27.4, *p* < 0.001). Pairwise analysis confirmed that Schirmer scores were significantly higher at all follow-up visits (T1, T3, T6 and T12) compared to baseline (T0) (*p* < 0.001), and a further significant improvement was observed between T1 and T6 (*p* = 0.003) and T3 and T12 (*p* < 0.001). Conjunctival hyperemia also improved significantly, with a Friedman test indicating a reduction in scores over time (χ^2^(4) = 34.5, *p* < 0.001). Pairwise comparisons demonstrated statistically significant decreases in hyperemia from baseline to each follow-up until T6 (*p* < 0.05), with additional improvements detected between T1 and T6 (*p* < 0.038), between T3 and T6 (*p* < 0.007), between T1 and T12 (*p* = 0.003) and between T3 and T12 (*p* < 0.001). In contrast, tear film stability, as measured by Tear Break-Up Time (TBUT), remained stable throughout the study period. Repeated measures ANOVA yielded no statistically significant differences in TBUT across visits (F(4) = 2.06, *p* = 0.1). Post hoc analysis after Bonferroni correction showed no statistical differences between each follow-up timeline. These findings are further detailed in Table 3 and illustrated in Figure 2, Figure 3 and Figure 4.

### 3.4. Glaucoma Indices

Comparative analysis between baseline (T0), the six-month follow-up (T6) and the twelve-month follow-up revealed no statistically significant deterioration in either functional or structural parameters associated with glaucoma progression. The mean change from baseline in Visual Field Index (VFI) was −1.08 ± 0.84, which did not reach statistical significance (*p* = 0.218). Similarly, MD showed a minor shift of 0.141 ± 0.018 dB (*p* = 0.15), while Pattern Standard Deviation (PSD) changed by 0.072 ± 0.09 dB (*p* = 0.39). Structural metrics assessed via OCT also remained stable, with the RNFL decreasing from baseline by −2.5 ± 1.99 µm (*p* = 0.11), and the GCC by −1.8 ± 3.72 µm (*p* = 0.18). These results, presented in Table 4, indicate that treatment with preservative-free travoprost did not contribute to measurable glaucomatous progression over the twelve-month observation period.

Based on the study results, it is evident that IOP is better controlled with travoprost compared to the previous treatment. Specifically, IOP values before treatment with travoprost showed a statistically significant difference compared to all values during follow-up (*p* < 0.01), while no difference was observed between measurements at T1, T3, and T6 time points (*p* > 0.05). The twelve-month follow-up showed significant difference between T1 and T3 follow-ups.

Regarding the severity parameters of ocular surface disease, clear improvement was observed in the Schirmer test and conjunctival hyperemia, while no improvement was seen in TBUT. Schirmer test at treatment initiation showed significant difference compared to all time points (T1–T12) (*p* < 0.01), with a difference also noted between T1 and T6 (*p* < 0.05) and T3 and T12 (*p* < 0.001). Conjunctival hyperemia improved significantly, with differences observed between baseline and all time points (T1–T12), as well as between T1 and T6, T3 and T6, T1 and T12 and T3 and T12 time points. The TBUT index showed no significant change during patients’ follow-up (*p* > 0.05).

As for the clinical indices of glaucoma progression during treatment, results indicate that preservative-free travoprost can effectively control glaucoma progression in patients with mild or moderate glaucoma, based on non-significant differences indicated through the follow-ups between VFI, MD, PSD, RNFL and GCC.

## 4. Discussion

The findings of this study demonstrate that preservative-free travoprost is effective in significantly lowering IOP in patients with mild to moderate POAG, aligning with the efficacy reported in earlier clinical trials. This reinforces its potential role as a reliable first-line treatment. Crucially, the therapeutic benefits were achieved without exacerbating OSD, a common concern with preserved formulations, particularly those containing BAK.

A particularly noteworthy result was the significant improvement in Schirmer test scores, suggesting enhanced tear production in patients treated with preservative-free travoprost. This is especially important for individuals predisposed to or already experiencing dry eye symptoms. The elimination of preservatives appears to minimize ocular surface stress, a theory further supported by the reduction in conjunctival hyperemia observed throughout the study. This aligns with the hypothesis that preservatives, particularly BAK, may induce low-grade inflammation and epithelial toxicity, exacerbating OSD during long-term topical therapy. The observed improvement in Schirmer test values indicates enhanced basal tear secretion, suggesting that the switch to preservative-free travoprost may help restore tear film homeostasis in patients with ocular surface compromise. This finding is clinically relevant, as increased tear production can alleviate dry eye symptoms, improve visual comfort, and support better adherence to long-term glaucoma therapy. Similarly, the significant reduction in conjunctival hyperemia reflects decreased ocular surface inflammation and irritation, both of which are frequently associated with preserved formulations. Together, these outcomes underscore the ocular surface benefits of preservative-free travoprost, contributing not only to patient tolerability but also to overall quality of life and treatment compliance.

While TBUT remained unchanged, this outcome may reflect the chronic and multifactorial nature of tear film instability, which is unlikely to resolve rapidly. The lack of TBUT improvement could also be influenced by the limitations of the test itself, which primarily assesses the lipid layer rather than the mucin layer-possibly the primary site of preservative-induced damage. The concurrent improvement in hyperemia despite stable TBUT suggests that underlying mucin-related dysfunction may have improved even if not fully captured by TBUT measurements [29].

Importantly, no statistically significant changes were observed in glaucoma indices, including VFI, MD, PSD, RNFL, GCC thickness during the 12-month follow-up period. These findings suggest that preservative-free travoprost maintained effective IOP control sufficient to preserve both structural and functional stability. Although the study duration may not have been long enough to detect subtle progressive changes, the absence of deterioration across these parameters indicates stable disease under treatment, supporting the efficacy and safety of preservative-free travoprost in maintaining glaucomatous stability.

An additional aspect that warrants consideration is the cost–benefit profile of preservative-free formulations. Although preservative-free prostaglandin analogues are generally associated with higher acquisition costs compared with preserved alternatives, these expenses may be offset by potential long-term savings. Preservatives such as BAK contribute to ocular surface disease, which frequently leads to increased healthcare utilization through the need for additional lubricants, anti-inflammatory treatments, and even surgical interventions in severe cases. Moreover, ocular discomfort and intolerance can negatively affect adherence to glaucoma therapy, increasing the risk of disease progression and associated costs. In patients with pre-existing ocular surface disease, the use of preservative-free therapy may therefore not only improve comfort and adherence but also reduce secondary treatment burden and long-term expenditure. By minimizing preservative-related ocular surface complications and improving patient tolerance, preservative-free formulations may enhance both quality of life and long-term cost-effectiveness of glaucoma management. Future pharmacoeconomic studies are needed to quantify these potential savings and further inform treatment decisions [30].

The present findings are consistent with prior studies examining preservative-free glaucoma therapies. Pisella et al. (2002), in a large observational study, found a significantly higher prevalence of OSD symptoms in patients using preserved medications [31]. Switching to preservative-free drops led to symptom improvement in most cases. Similarly, Oddone et al. (2022) demonstrated that patients switching to preservative-free tafluprost/timolol experienced both improved IOP control and reduced OSD signs [32]. Jandroković et al. (2022) also reported improved ocular surface integrity and reduced hyperemia following a similar therapeutic switch [33]. Although other studies, such as those by Shedden et al. (2010) and Day et al. (2013), found comparable efficacy and tolerability between preserved and preservative-free treatments, the trend across multiple investigations supports a favorable safety profile for preservative-free agents, particularly in patients with ocular surface sensitivity [34,35].

Goldberg et al. in 2014 compared the efficacy of two bimatoprost/timolol formulations (with and without preservatives) [36]. A total of 561 patients with primary open-angle glaucoma and ocular hypertension were randomly assigned in this study. The study results showed the non-inferiority of the preservative-free formulation compared to the preservative-containing one in IOP control, with no significant differences observed in conjunctival hyperemia and punctate keratitis [36].

Aihara et al. (2012) provided long-term evidence for BAK-free travoprost, showing sustained IOP reduction and notable improvements in corneal and conjunctival health [27]. Collectively, the current study adds to a growing body of evidence suggesting that while preservative-free medications perform comparably to preserved ones in IOP control, they may offer superior ocular surface tolerability. Nevertheless, discrepancies in the literature highlight the need for larger, randomized, long-term studies directly comparing these formulations under standardized conditions.

Kumar et al. (2020) compared BAK-preserved travoprost with BAKfree travoprost in Indian glaucoma patients and reported that the BAC-free formulation was associated with significantly fewer ocular surface disease symptoms and better quality of life [37]. Ruangvaravate et al. (2020) studied patients switching from other prostaglandins to tafluprost or preservative-free tafluprost and demonstrated improvements in ocular surface parameters, with the preservative-free formulation providing the greatest benefit [38]. Muz et al. (2021) [39] compared BAK-preserved latanoprost with polyquad-preserved travoprost and observed superior ocular surface outcomes with the polyquad-preserved formulation, while intraocular pressure reduction remained comparable between groups. These findings are consistent with the present study, which further extends the literature by specifically evaluating a completely preservative-free travoprost formulation. Unlike formulations containing alternative preservatives such as polyquad, our results suggest that the elimination of all preservatives may provide additional advantages for ocular surface health, while maintaining robust efficacy in IOP control [37,38,39].

As mentioned above, our findings are consistent with yet expand upon, the growing body of evidence supporting the efficacy and tolerability of preservative-free prostaglandin analogues. Early studies, such as those by Pisella et al. (2002), demonstrated significant improvement in ocular surface symptoms following a switch from preserved to preservative-free glaucoma medications, while Oddone et al. (2022) and Jandroković et al. (2022) further confirmed improvements in ocular surface integrity and reduction in hyperemia with preservative-free tafluprost/timolol combinations [31,32,33]. These observations have been reinforced by more recent large-scale and phase III clinical trials.

Baudouin et al. (2025) conducted a phase III multicentre trial comparing preservative-free latanoprost emulsion with a preserved formulation, demonstrating non-inferiority in IOP control while achieving significantly better tolerability and fewer ocular surface adverse events [40]. Similarly, Czumbel et al. (2024) confirmed the therapeutic equivalence and enhanced comfort of preservative-free generic latanoprost compared with the preserved reference drug (Xalatan^®^, Pfizer, Puurs, Belgium) [41]. Brinkman et al. (2024) reported improved tolerability profiles with preservative-free tafluprost compared to preserved latanoprost, while maintaining comparable IOP-lowering efficacy [42]. Consistent findings were observed in the COMfort Eye Trial (COMET) by Kandarakis et al. (2024), which confirmed the non-inferiority of preservative-free latanoprost versus Xalatan^®^ in patients with POAG or OHT, alongside a significant reduction in ocular surface discomfort [43]. Collectively, these studies confirm that removing preservatives enhances ocular surface safety without compromising antiglaucoma efficacy. The safety of preservative-free ophthalmic formulations has been widely discussed in the literature, as preservatives such as benzalkonium chloride (BAK) are known to induce or exacerbate ocular surface alterations, including epithelial toxicity, tear film instability, and ocular surface inflammation. Several studies have reported improved ocular surface tolerance with preservative-free formulations in comparison with their preserved counterparts [25,26,27].

Regarding prostaglandin analogues, earlier investigations primarily focused on BAK-free travoprost formulations, in which alternative preservatives (e.g., polyquaternium-1, SofZia^®^) were used. These studies consistently demonstrated a more favorable ocular surface profile than BAK-preserved travoprost, but they did not evaluate a formulation completely free of preservatives [25,26,27].

To our knowledge, the present study is the first to specifically assess the impact of a preservative-free travoprost formulation on the ocular surface. This distinction is important, as even alternative preservatives may exert subclinical or long-term effects on the ocular surface. By eliminating preservatives altogether, preservative-free travoprost offers a potentially safer therapeutic option for patients at risk of ocular surface disease, such as those with concomitant dry eye or requiring long-term intraocular pressure–lowering therapy.

Our findings add to the existing body of evidence by demonstrating that preservative-free travoprost maintains efficacy in intraocular pressure control while minimizing ocular surface compromise. This is particularly relevant for glaucoma patients who often require chronic therapy, in whom ocular surface health is a critical determinant of treatment adherence and quality of life.

Despite these promising findings, the present study has certain limitations. The absence of objective biomarkers for ocular surface inflammation and the reliance on clinical grading scales may have restricted the sensitivity of surface assessments. A limitation of the present study is the absence of blinding, as it was conducted in an open-label design. Although standardized assessments by a single examiner reduced variability, some degree of observer bias cannot be excluded. An additional consideration is that our study population included both treatment-naïve patients and those who had previously experienced poor tolerability with preserved antiglaucoma therapies. While combining these subgroups follows the methodology of prior studies [25,26,27], it may have masked potential differences in response or tolerability between them. We acknowledge this as a limitation and emphasize that larger studies with sufficient statistical power are needed to perform separate analyses of these clinically relevant subgroups. Future studies incorporating randomized control groups, longer follow-up periods, and objective surface evaluation methods with patient-report outcome using quality of life questionnaires are warranted to validate and expand upon these findings, assess the sustained impact on ocular surface health, and determine whether the observed short-term benefits translate into long-term disease stability and improved patient outcomes. Missing data were handled using a complete-case approach, as the proportion of dropouts was low. It is acknowledged that sensitivity analyses, such as intention-to-treat-based methods, could further assess the robustness of our findings and should be incorporated in future larger, randomized studies to minimize bias related to loss-to-follow-up.

The findings of this study have meaningful implications for clinical practice, especially in patients with preexisting OSD. Given that these patients are more susceptible to preservative-induced epithelial toxicity and inflammation, the use of preservative-free travoprost offers a safer and more tolerable therapeutic alternative. Improved ocular surface comfort and reduced hyperemia can enhance treatment adherence and overall quality of life—key factors in successful long-term glaucoma management. Therefore, preservative-free prostaglandin formulations should be strongly considered as first-line therapy, including other preservative-free formulation with the same efficacy, for patients with OSD or those requiring chronic topical treatment.

## 5. Conclusions

In conclusion, preservative-free travoprost offers an effective and well-tolerated treatment for patients with primary open-angle glaucoma, reducing IOP while minimizing the risk of ocular surface disease. The absence of preservatives like BAK in travoprost appears to alleviate many of the adverse effects associated with long-term glaucoma treatment.

## Figures and Tables

**Table 1:** Demographic Data.

Parameters	
Age (years, Median [IQR])	75 [73, 82]
Gender (*n*)	22 male, 17 female
Duration of disease (years, Median [IQR])	6.5 [1, 15]
Previous medication (*n*)	15 latanoprost, 13 tafluprost

IQR—interquartile range.

**Table 2:** IOP comparisons.

Follow-Up	Number of Patients	Median (mmHg) [IQR]	*p* < 0.05 (Mean Difference)	*p*-Value
IOP Τ0	39	18 [15, 20]	IOP Τ1 (3)	*p* < 0.001
			IOP Τ3 (2)	*p* < 0.001
			IOP T6 (3)	*p* < 0.001
IOP Τ1	39	15 [14, 16]	IOP Τ0	*p* < 0.001
IOP Τ3	39	16 [14.75, 17]	IOP Τ0	*p* < 0.001
IOP Τ6	39	15 [13.5, 15]	IOP Τ0	*p* < 0.001
IOP T12	39	14 [12.8, 15]	IOP T0 (4)	*p* < 0.001
			IOP T1 (3)	*p* = 0.007
			IOP T3 (2)	*p* < 0.001

IQR—interquartile range. IOP—intraocular pressure.

**Table 3:** Ocular surface disease parameters comparison.

Follow-Up	Number of Patients	Median [IQR]	Statistically Significant Difference Between the Time Points (*p* < 0.05) (Mean Difference)	*p*-Values
Schirmer test Τ0 (mm)	39	7 [5, 12.25]	Schirmer Τ1 (3)	*p* < 0.001
			Schirmer Τ3 (5.5)	*p* < 0.001
			Schirmer Τ6 (5.5)	*p* = 0.013
Schirmer test Τ1 (mm)	39	10 [6, 15]	Schirmer T0(3)	*p* < 0.001
			Schirmer T6 (2.5)	*p* = 0.013
Schirmer test Τ3 (mm)	39	12.5 [10, 15.5]	Schirmer T0 (5.5)	*p* < 0.001
Schirmer test Τ6 (mm)	39	12.5 [10, 16.5]	Schirmer T0 (5.5)	*p* < 0.001
			Schirmer T1 (2.5)	*p* = 0.013
Schirmer test Τ12 (mm)	39	13.5 [11, 17.3]	Schirmer T0 (6.5)	*p* < 0.001
			Schirmer T1 (3.5)	*p* < 0.001
			Schirmer T3 (1)	*p* < 0.001
TBUT T0 (s)	39	14 [10, 19.5]	N/A	*p* > 0.05
TBUT T1 (s)	39	16 [11.5, 19]	N/A	*p* > 0.05
TBUT T3 (s)	39	16 [14.5, 20]	N/A	*p* > 0.05
TBUT T6 (s)	39	16,5 [14, 20]	N/A	*p* > 0.05
TBUT T12 (s)	39	17 [15, 21]	N/A	*p* > 0.05
CH Τ0	39	2 [1, 2]	CH Τ1	*p* = 0.005
			CH Τ3	*p* = 0.027
			CH Τ6	*p* < 0.001
CH Τ1	39	1 [1, 2]	CH Τ0	*p* = 0.005
			CH Τ6	*p* = 0.038
CH Τ3	39	1 [1, 2]	CH Τ0	*p* = 0.027
			CH Τ6	*p* = 0.007
CH Τ6	39	1 [1, 2]	CH Τ0	*p* < 0.001
			CH Τ1	*p* = 0.038
			CH Τ3	*p* = 0.007
CH T12	39	1 [1, 2]	CH Τ0	*p* < 0.001
			CH Τ1	*p* = 0.003
			CH Τ3	*p* < 0.001

IQR—interquartile range. TBUT—tear break-up time. CH—conjunctival hyperemia.

**Table 4:** Glaucoma indices comparison.

Glaucoma Indices	Number of Patients	T0 (Mean ± SD)	T6 (Mean ± SD)	T12 (Mean ± SD)	Mean Difference (from Baseline) ± SD [95% CI]	*p*-Values
VFI (%)	39	84.55 ± 23.86	82.83 ± 24.26	82.4 ± 24.35	−1.083 ± 0.84 [−0.62, 2.79]	*p* = 0.218
MD (dB)	39	−6.54 ± 8.38	−6.66 ± 8.40	−6.69 ± 8.51	0.141 ± 0.018 [0.1, 0.177]	*p* = 0.15
PSD (dB)	39	3.88 ± 3.06	3.93 ± 3.33	3.95 ± 3.13	0.072 ± 0.095 [−0.245, 0.099]	*p* = 0.39
RNFL (μm)	39	87.17 ± 9.76	85 ± 8.38	84.4 ± 9.54	−2.5 ± 1.99 [−1.25, −1.96]	*p* = 0.11
GCC (μm)	39	89.68 ± 11.3	88.4 ± 11.31	87.9 ± 11.2	−1.8 ± 3.72 [−2.35, 0.15]	*p* = 0.18

VFI—visual field index. MD—mean deviation. PSD—pattern standard deviation. RNFL—retinal nerve fiber layer. GCC—ganglion cell complex. dB—decibel. μm—micrometers.

## Data Availability

The data supporting the findings of this study are available from the corresponding author upon reasonable request. Due to ethical and privacy considerations, certain restrictions may apply to the availability of the data.

## Abbreviations

The following abbreviations are used in this manuscript:

IOP	Intraocular Pressure
POAG	Primary Open-Angle Glaucoma
OHT	Ocular Hypertension
BAK	Benzalkonium Chloride
OSD	Ocular Surface Disease
TBUT	Tear Break-Up Time
CCT	Central Corneal Thickness
OCT	Optical Coherence Tomography
RNFL	Retinal Nerve Fiber Layer
GCC	Ganglion Cell Complex
VFI	Visual Field Index
MD	Mean Deviation
PSD	Pattern Standard Deviation
ANOVA	Analysis of Variance
CH	conjunctival hyperemia
SITA	Swedish Interactive Threshold Algorithm
IQR	Interquartile Range
SD	Standard Deviation

## References

[1] European Glaucoma Society (2021). Terminology and Guidelines for Glaucoma, 5th ed. Br. J. Ophthalmol..

[2] Quigley H.A., Broman A.T. (2006). The number of people with glaucoma worldwide in 2010 and 2020. Br. J. Ophthalmol..

[3] Kang J.M., Tanna A.P. (2021). Glaucoma. Med. Clin..

[4] Prum B.E., Rosenberg L.F., Gedde S.J., Mansberger S.L., Stein J.D., Moroi S.E., Herndon L.W., Lim M.C., Williams R.D. (2016). Primary Open-Angle Glaucoma Preferred Practice Pattern^®^ Guidelines. Ophthalmology.

[5] Gedde S.J., Vinod K., Wright M.M., Muir K.W., Lind J.T., Chen P.P. (2021). Primary Open-Angle Glaucoma Preferred Practice Pattern^®^. Ophthalmology.

[6] Gedde S.J., Lind J.T., Wright M.M., Chen P.P., Muir K.W., Vinod K., Li T., Mansberger S.L. (2021). Primary Open-Angle Glaucoma Suspect Preferred Practice Pattern^®^. Ophthalmology.

[7] Moshegov S., Kerr N.M. (2023). Prostaglandin FP receptor agonists in the treatment of glaucoma and ocular hypertension: A literature review. Expert. Opin. Investig. Drugs.

[8] Arranz-Marquez E., Teus M.Á. (2022). Prostaglandin analogues for the treatment of glaucoma: From the ‘wonder’ drug of the ‘90s to the reality of the 21st century. Arch. Soc. Esp. Oftalmol..

[9] Matsou A., Anastasopoulos E. (2018). Investigational drugs targeting prostaglandin receptors for the treatment of glaucoma. Expert Opin. Investig. Drugs.

[10] Mohan N., Chakrabarti A., Nazm N., Mehta R., Edward D.P. (2022). Newer advances in medical management of glaucoma. Indian J. Ophthalmol..

[11] Zhang X.L., Qin L. (2019). Efficacy of travoprost for the treatment of patients with glaucoma. Medicine.

[12] Li T., Lindsley K., Rouse B., Hong H., Shi Q., Friedman D.S., Wormald R., Dickersin K. (2016). Comparative effectiveness of first-line medications for primary open-angle glaucoma: A systematic review and network meta-analysis. Ophthalmology.

[13] Peace J.H., Ahlberg P., Wagner M., Lim J.M., Wirta D., Branch J.D. (2015). Polyquaternium-1-preserved travoprost 0.003% or benzalkonium chloride-preserved travoprost 0.004% for glaucoma and ocular hypertension. Am. J. Ophthalmol..

[14] Goldstein M.H., Silva F.Q., Blender N., Tran T., Vantipalli S. (2022). Ocular benzalkonium chloride exposure: Problems and solutions. Eye.

[15] Kahook M.Y., Rapuano C.J., Messmer E.M., Radcliffe N.M., Galor A., Baudouin C. (2024). Preservatives and ocular surface disease: A review. Ocul. Surf..

[16] Kaštelan S., Tomić M., Soldo K.M., Salopek-Rabatić J. (2013). How ocular surface disease impacts the glaucoma treatment outcome. BioMed Res. Int..

[17] Steven D.W., Alaghband P., Lim K.S. (2018). Preservatives in glaucoma medication. Br. J. Ophthalmol..

[18] Kemer Ö.E., Mekala P., Dave B., Kooner K.S. (2024). Managing ocular surface disease in glaucoma treatment: A systematic review. Bioengineering.

[19] Konstas A.G., Labbé A., Katsanos A., Meier-Gibbons F., Irkec M., Boboridis K.G., Holló G., García-Feijoo J., Dutton G.N., Baudouin C. (2021). The treatment of glaucoma using topical preservative-free agents: An evaluation of safety and tolerability. Expert. Opin. Drug Saf..

[20] Riedlová K., Saija M.C., Olżyńska A., Jurkiewicz P., Daull P., Garrigue J.S., Cwiklik L. (2023). Influence of BAKs on tear film lipid layer: In vitro and in silico models. Eur. J. Pharm. Biopharm..

[21] Baudouin C., Labbé A., Liang H., Pauly A., Brignole-Baudouin F. (2010). Preservatives in eyedrops: The good, the bad and the ugly. Prog. Retin. Eye Res..

[22] Sanford M. (2014). Preservative-free latanoprost eye drops in patients with primary open-angle glaucoma/ocular hypertension. Clin. Drug Investig..

[23] Harasymowycz P., Hutnik C., Rouland J.-F., Negrete F.J.M., Economou M.A., Denis P., Baudouin C. (2021). Preserved versus preservative-free latanoprost for the treatment of glaucoma and ocular hypertension: A post hoc pooled analysis. Adv. Ther..

[24] Kim K.E., Lee C.K., Shin J., Kim Y., Rho S. (2023). Comparisons of efficacy and safety between preserved and preservative-free brimonidine tartrate in glaucoma and ocular hypertension: A parallel-grouped, randomized trial. Sci. Rep..

[25] Henry J.C., Peace J.H., Stewart J.A., Stewart W.C. (2008). Efficacy, safety, and improved tolerability of travoprost BAK-free ophthalmic solution compared with prior prostaglandin therapy. Clin. Ophthalmol..

[26] García-Feijoo J., Muñoz-Negrete F.J., Hubatsch D.A., Rossi G.C. (2016). Efficacy and tolerability of benzalkonium chloride-free travoprost in glaucoma patients switched from benzalkonium chloride-preserved latanoprost or bimatoprost. Clin. Ophthalmol..

[27] Aihara M., Otani S., Kozaki J., Unoki K., Takeuchi M., Minami K., Miyata K. (2012). Long-term effect of BAK-free travoprost on ocular surface and intraocular pressure in glaucoma patients after transition from latanoprost. J. Glaucoma.

[28] Efron N., Morgan P.B., Katsara S.S. (2001). Validation of grading scales for contact lens complications. Ophthalmic Physiol. Opt..

[29] Ramli N., Supramaniam G., Samsudin A., Juana A., Zahari M., Choo M.M. (2015). Ocular surface disease in glaucoma: Effect of polypharmacy and preservatives. Optom. Vis. Sci..

[30] Canut M.I., García-Feijoo J., Larrosa-Poves J.M., López-López F., Pazos M., Espinoza-Cámac N., Oyagüez I., Del Rio T., Rodríguez M. (2025). Cost-utility analysis of latanoprost unidose cationic emulsion preservative-free versus latanoprost unidose in the treatment of open-angle glaucoma and ocular hypertension patients in Spain. Expert Rev. Pharmacoeconomics Outcomes Res..

[31] Pisella P.J., Pouliquen P., Baudouin C. (2002). Prevalence of ocular symptoms and signs with preserved and preservative free glaucoma medication. Br. J. Ophthalmol..

[32] Oddone F., Kirwan J., Lopez-Lopez F., Zimina M., Fassari C., Holló G. (2022). Switching to preservative-free tafluprost/timolol fixed-dose combination in the treatment of open-angle glaucoma or ocular hypertension: Subanalysis of data from the VISIONARY Study according to baseline monotherapy treatment. Adv. Ther..

[33] Jandroković S., Pauk S.V., Gaćina D.L., Skegro I., Tomić M., Masnec S., Kuzman T., Kalauz M. (2022). Tolerability in glaucoma patients switched from preserved to preservative-free prostaglandin-timolol combination: A prospective real-life study. Clin. Ophthalmol..

[34] Shedden A., Adamsons I.A., Getson A.J., Laurence J.K., Lines C.R., Hewitt D.J., Ho T.W. (2010). Comparison of the efficacy and tolerability of preservative-free and preservative-containing formulations of the dorzolamide/timolol fixed combination (COSOPT™) in patients with elevated intraocular pressure in a randomized clinical trial. Graefes Arch. Clin. Exp. Ophthalmol..

[35] Day D.G., Walters T.R., Schwartz G.F., Mundorf T.K., Liu C., Schiffman R.M., Bejanian M. (2013). Bimatoprost 0.03% preservative-free ophthalmic solution versus bimatoprost 0.03% ophthalmic solution (Lumigan) for glaucoma or ocular hypertension: A 12-week, randomised, double-masked trial. Br. J. Ophthalmol..

[36] Goldberg I., Gil Pina R., Lanzagorta-Aresti A., Schiffman R.M., Liu C., Bejanian M. (2014). Bimatoprost 0.03%/timolol 0.5% preservative-free ophthalmic solution versus bimatoprost 0.03%/timolol 0.5% ophthalmic solution (Ganfort) for glaucoma or ocular hypertension: A 12-week randomised controlled trial. Br. J. Ophthalmol..

[37] Kumar S., Singh T., Ichhpujani P., Vohra S., Thakur S. (2020). Correlation of ocular surface disease and quality of life in Indian glaucoma patients: BAC-preserved versus BAC-free travoprost. Turk. J. Ophthalmol..

[38] Ruangvaravate N., Choojun K., Srikulsasitorn B., Chokboonpiem J., Asanatong D., Trakanwitthayarak S. (2020). Ocular surface changes after switching from other prostaglandins to tafluprost and preservative-free tafluprost in glaucoma patients. Clin. Ophthalmol..

[39] Muz O.E., Dagdelen K., Pirdal T., Guler M. (2021). Comparison of BAK-preserved latanoprost and polyquad-preserved travoprost on ocular surface parameters in patients with glaucoma and ocular hypertension. Int. Ophthalmol..

[40] Baudouin C., Stalmans I., Bourne R., Larrosa J.M., Schmickler S., Seleznev A., Oddone F., Phase III study group (2025). A phase III study comparing preservative-free latanoprost eye drop emulsion with preserved latanoprost in open-angle glaucoma or ocular hypertension. Eye.

[41] Czumbel N., Acs T., Bator G., Halmosi A., Egorov E.A., Maltsev D.S. (2024). A phase III, multicentre, randomised, investigator-masked, cross-over, comparative, non-inferiority trial evaluating the efficacy and tolerability of generic preservative-free Latanoprost (Polpharma, S.A.) compared to Xalatan^®^ (Pfizer) in patients with ocular hypertension or primary open-angle glaucoma. BMC Ophthalmol..

[42] Brinkman D., McSwiney T., James M. (2024). Comparing the tolerability of preservative-free tafluprost versus preserved latanoprost in the management of glaucoma and ocular hypertension—An observer blinded active-control trial. Ir. J. Med. Sci..

[43] Kandarakis S., Papadopoulos A.P., Roussopoulos G., Georgopoulos E., Chung Y., Doumazos L., Baek A., Paizi N.I., Shin H., Papadopoulos P.A. (2024). COMfort Eye Trial (COMET) results—A non-inferiority, randomized, investigator-masked, two-parallel group, phase III clinical trial, to evaluate the efficacy and safety of a preservative free formulation of latanoprost versus a reference drug (Xalatan^®^) in patients with primary open-angle glaucoma (POAG) or ocular hypertension (OHT). Expert Opin. Drug Saf..

